# A forgotten disease: Pelvic congestion syndrome as a cause of chronic lower abdominal pain

**DOI:** 10.1371/journal.pone.0213834

**Published:** 2019-04-02

**Authors:** Agnieszka Jurga-Karwacka, Grzegorz M. Karwacki, Andreas Schoetzau, Christoph J. Zech, Viola Heinzelmann-Schwarz, Fabienne D. Schwab

**Affiliations:** 1 Department of Gynecology and Gynecological Oncology, University Women`s Hospital of Basel, University of Basel, Basel, Switzerland; 2 Clinic of Radiology & Nuclear Medicine, University Hospital of Basel, University of Basel, Basel, Switzerland; 3 Ovarian Cancer Research, Department of Biomedicine, University of Basel, Basel, Switzerland; University Magna Graecia of Catanzaro, ITALY

## Abstract

**Objectives:**

Pelvic congestion syndrome is defined as chronic pelvic pain due to incompetent (dilated and refluxing) pelvic veins. The aim of this study was to investigate the prevalence of this condition by examining the prevalence of dilated ovarian and para-uterine veins in pre- and postmenopausal female patients, irrespective of their symptoms. We subsequently investigated how many women with dilated veins suffered from chronic pelvic pain. Additionally, we attempted to define diagnostic criteria that may allow for early identification of affected patients.

**Methods:**

We reassessed 2384 abdomino-pelvic computed tomography scans performed on women at our institution. The maximal diameters of the ovarian and para-uterine veins were measured. Patients with a pathological process in the abdomen or pelvis affecting the veins were excluded. We considered ovarian vein dilation to be 6 mm or more in the axial plane. For patients that met these criteria, we performed a retrospective chart review to evaluate the clinical presentation and/or symptoms of these patients.

**Results:**

Dilated pelvic veins were present in 293/2384 (12%) patients, 118/559 premenopausal (21%) and 175/1825 postmenopausal (10%). Chronic pelvic pain of unclear etiology had been documented prior to the CT in 54/293 (18%) women with dilated veins—2% of the whole study collective (54/2384); 8% of all premenopausal (44/559) and 0.5% of all postmenopausal (10/1825). It was often accompanied by urological symptoms such as hematuria, dysuria, and urinary frequency, in the absence of infection (p<0.05). We identified a strong correlation between the presence of dilated ovarian veins and chronic pelvic pain in premenopausal parous patients with hematuria.

**Conclusions:**

Pelvic congestion syndrome appears to be an underdiagnosed and undertreated disease. In our study, 8% of all premenopausal women had documented chronic pelvic pain of unclear etiology and dilated ovarian and pelvic veins on cross-sectional imaging studies. The features we identified in this study as most relevant should enable a faster identification of patients who could benefit from a specific treatment regimen for this condition.

## Introduction

Pelvic congestion syndrome (PCS) is a condition that results from incompetent pelvic veins, causing chronic pelvic pain (CPP) in women. The indicators of incompetent veins comprise dysfunctional dilatation of ovarian (OV) and para-uterine veins (PV), slow blood flow (congestion), retrograde flow and reflux [1].

PCS was first described in 1949 as symptoms of CPP related to dilated pelvic veins [2,3]. According to the literature, women with PCS-derived CPP are mostly multiparous and describe the abdominal pain as dull and achy with sharp exacerbations, getting worse after long periods of standing and walking [1]. The pain can be accompanied by other symptoms such as post-coital pelvic aching and ovarian point tenderness on bimanual examination (94% sensitivity and 77% specificity) [4]. Dysuria, urinary frequency and urgency in women with unremarkable urine samples have also been described [5], as well as vulvar and lower extremity varicosities with lower limb chronic venous insufficiency [6,7]. The etiology of PCS is still poorly understood but likely multifactorial, involving both mechanical and hormonal factors.

Identification of dilated and dysfunctional pelvic veins is crucial for the diagnosis. The gold standard in this field is digital subtraction venography, but comparable results can be obtained by non-invasive magnetic resonance venography [6], computed tomography (CT) and Doppler sonography [8,9]. During diagnostic laparoscopy, dilated pelvic veins can be easily overlooked [10].

There are many treatment options for PCS. One of the best established is endovascular occlusion of the dilated vessels [11,12].

PCS may contribute up to 30% of all CPP referrals. Multiple studies have investigated this condition, including its diagnostic and therapeutic options. However, due to a lack of methodological consistency and robust data, the optimal diagnostic algorithm for PCS remain controversial [13,14].

CPP is defined as constant or recurring, cyclical or non-cyclical pain in the lower abdomen or pelvis of a minimum of 6 months duration, causing functional disability or limitation in activities of daily living [13]. In the UK, the incidence of CPP is 38/1000 women annually, comparably common as asthma (37/1000) and back pain (41/1000) [3]. Its socio-economic implications become more apparent when considering that approximately 10% of all gynecological referrals and operations are done for CPP [13,14].

It could therefore be assumed that PCS is a commonly encountered gynecological problem in daily clinical practice. However, it remains a scarcely recognized condition. Previously published data on the prevalence of symptomatic and asymptomatic pelvic varicosities have included no more than 324 patients [15,16,17]. Although these findings have been quoted in numerous publications, no further studies have attempted to confirm the results in a larger cohort. Additionally, the menopausal status of patients has not previously been taken into consideration in the evaluation of PCS prevalence [18].

The aim of this study was to retrospectively investigate the prevalence of PCS. We measured the maximal diameter of OV and PV in premenopausal (PreMP) and postmenopausal women (PMP) and the proportion of those with dilated veins that suffered from CPP. Furthermore, we attempted to establish standardized diagnostic criteria, based on medical history and imaging, to enable physicians to identify patients who could benefit from further diagnostic evaluation for this elusive condition.

## Methods

We retrospectively re-assessed all abdominopelvic CT examinations performed on women in the radiology department of the University Hospital of Basel from 01.01.2014 until 30.06.2014, irrespective of the indication or referring department. Patients were examined on 64-row and 128-row multidetector CT scanners (Somatom Emotion 16, Somatom Definition Flash and Somatom Definition, AS+, Siemens, Forchheim, Germany). Contrast medium (Ultravist 370, Bayer Schering Pharma AG, Berlin, Germany) was administered in an antecubital vein using a power injector. An amount of 80 to 100 ml contrast medium was injected at a rate of 2 ml/s followed by a 50ml saline flush at a rate of 2 ml/s. Individual contrast enhancement during the portal venous phase was optimized by applying real-time bolus tracking (Care Bolus, Siemens).

During the examination, patients performed a breath hold or a moderate forced attempted exhalation against a closed airway (Valsalva maneuver).

Image review was performed on PACS workstations (Centricity, GE Healthcare, USA). Two independent observers (AJK, GMK) investigated the scans in order to evaluate the largest diameters of the left and right OV, as well as the most dilated PV on each side. Following previously published results, we considered the OV to be dilated when measuring 6 mm or more in the axial plane [8,16,17,19,20,21,22]. Furthermore, we documented any anatomical (nutcracker syndrome, inferior vena cava duplication, May-Thurner syndrome) impairments of pelvic venous blood flow. Patients with intra-abdominal pathologies resulting in renal, ovarian, iliac or pelvic venous, as well as vena cava compression, as well as those with incomplete depiction of OV, were excluded from the study (Table 1). All measurements and interpretations were performed independently by a board-approved radiologist (GMK) and a resident of Obstetrics and Gynecology (AJK) trained in CT body imaging.

**Table 1:** Exclusion criteria for CT assessment.

Peritoneal carcinomatosis Pronounced retroperitoneal lymphadenopathy, fibrosis, tumor, aortic aneurysm or dissection with renal or iliac vein, or vena cava compression Neoplastic disease of kidneys, colon, rectum, ovaries, endometrium or cervix Ovarian, renal, portal vein or vena cava thrombosis Pelvic inflammatory disease Excessive ascites Renal trauma Pronounced uterine fibroids Early postoperative CT examination Incomplete depiction of ovarian veins

We subsequently retrospectively reviewed the medical records of the patients with dilated OV and the control group without dilated OV, including gynecological examinations available either in electronic medical records (ISMed, ProtecData AG, Switzerland) or matching paper-based gynecological files accessible within the hospital records. PCS-associated symptoms of interest included chronic or recurrent abdominal, back and flank pain, dyspareunia, ovarian point tenderness on bimanual examination, urological symptoms (dysuria, urinary frequency, urgency, hematuria), and vulvar and lower extremity varicosities (Table 2). Demographic information was also collected: menopausal status, age, ethnicity, height, body mass index (BMI), blood group, gravidity, parity, birth weights of children, method of contraception, position of uterus (ante- or retroverted), smoking, alcohol consumption and signs of infection as measured in blood tests (CPR, leucocytes). In women in whom menopausal status was not documented, we used age to assign them to a group (<50 years—premenopausal) [23].

**Table 2:** Documented symptoms.

Chronic or recurrent abdominal pain	
- localization:	whole abdomen lower abdomen flanks back
- side:	left right
- character:	constant colicky stubbing dull
Dyspareunia Urological symptoms - hematuria - dysuria - urinary frequency - urgency Ovarian point tenderness	
Varicosis	
- localization:	lower extremities vulva anus
- side:	right left

For all analyses, patients with documented chronic pelvic pain of no specific etiology and dilated OV (≥ 6 mm) were suspected to have PCS. Women with dilated OV but no CPP as well as women with neither vein-dilatation nor CPP were considered as the two control groups.

All the patient records were fully anonymized. Statistical analysis was made using the computer software R (R Development Core Team, 2015). In order to compare the study and control groups, T-tests were used for metric variables and Fisher's exact Test was applied for categorical variables. To select best predictors for study groups, a conditional regression tree was performed. Results are presented as a diagram with the selected "best" variables and corresponding p-values. Detailed description of the tree-based model is given elsewhere [24]. A *P* value of <0.05 was considered as significant. All measurements were independently performed by two examiners with good inter-observer strength of agreement (κ = 0.74).

The study was performed with ethical approval of the institutional review board (Ethics Committee of northwest/central Switzerland 2015–129). Because of the retrospective character of the study the ethics committee waived the requirement for informed consent.

## Results

During the study period, 2402 women underwent abdominopelvic CT examination. We excluded 18 cases in which either the visualization of OV was incomplete or the vascular dilation was possibly due to a pathological process in the pelvis (Table 1). Among the remaining 2384 women (559 PreMP, mean age 37±8 years; 1825 PMP, mean age 70±12 years), dilated OV with or without dilated PV were found in 293 (12%) patients. These patients comprised our study group.

All 293 women, 118/559 PreMP (21%) and 175/1825 PMP (10%), had an electronic patient file and 163 of them had a specific gynecological chart in our outpatient clinic. CPP of unclear etiology was documented in 54/293 (18%) cases with dilated OV, 44/118 PreMP (37%) and 10/175 PMP (6%). The prevalence of symptomatic dilated OV (PCS) in the whole study collective (n = 2384) was 2% but reached 8% in the PreMP (n = 559) and was 0.5% in the PMP (n = 1825).

Asymptomatic dilated OV with or without PV were found in 239/2384 (10%) patients, 74/559 PreMP (13%) and 165/1825 PMP (9%), and comprised our first control group (CG1). From the 2090 patients without dilated OV, we randomly chose 103 healthy women (age-matched to our study group) for our second control group (CG2), which was defined as women with neither venous dilatation nor CPP.

There was a statistically significant difference between the PCS patients and the control groups in terms of age (p<0.001), menopausal status (p<0.001), ethnicity (p<0.01), character of pain (p<0.001), accompanying symptoms like hematuria (p<0.001), dysuria (p<0.01) and urinary frequency (p<0.03, Table 3). The PCS patients in our study were mostly premenopausal (81%), in their late thirties or early forties (mean age 40.1 ± 10.5 years) and over 40% were of Mediterranean ethnicity. They reported chronically recurring colicky pain, localized in the lower abdomen, back and flanks, which was commonly associated with urological symptoms such as hematuria, dysuria and urinary frequency in the absence of infection (p<0.05, respectively). Nutcracker syndrome, a vascular compression disorder that refers to the compression of the left renal vein between the proximal superior mesenteric artery and the abdominal aorta, was the cause of symptomatic OV-dilatation in 5 women. Other vascular disorders such as duplication of the inferior vena cava or May-Thurner syndrome were not found in our study collective.

**Table 3:** PCS associated characteristics.

	PCSG n = 54	CG1 n = 239	P-value (PCS vs. CG1)	CG2 n = 103	P-value (PCS vs. CG2)
Age (SD)	40.1 (10.5)	57.5 (17.2)	<0.001	43.2 (16.9)	0.05
Premenopausal status (%)	44 (81)	73 (30)	<0.001	70 (68)	0.05
Mediterranean ethnicity (%)	22 (41)	44 (18)	0.01	19 (18)	0.01
Lower abdominal pain, left (%)	32 (59)	76 (32)	0.002	25 (24)	<0.001
Lower abdominal pain, right (%)	34 (63)	70 (29)	<0.001	26 (25)	<0.001
Back pain (%)	17 (31)	22 (9)	0.001	9 (9)	0.001
Flank pain, left (%)	24 (44)	50 (21)	0.005	17 (16)	0.001
Flank pain, right (%)	19 (35)	42 (17)	0.03	12 (12)	0.003
Colic (%)	25 (46)	24 (10)	<0.001	12 (12)	<0.001
Hematuria (%)	21 (39)	28 (12)	<0.001	7 (7)	<0.001
Dysuria (%)	13 (24)	15 (6)	0.01	7 (7)	0.01
Urinary frequency (%)	9 (17)	9 (4)	0.03	5 (5)	0.03

PCS: pelvic congestion syndrome

PCSG: pelvic congestion syndrome group

CG1: control group 1 (dilated ovarian or para-uterine veins, no chronic pelvic pain)

CG2: control group 2 (normal ovarian and para-uterine veins, no chronic pelvic pain)

SD: standard deviation

There was no statistically significant difference between the PCS group and the controls in terms of previously reported gravidity and parity (p>0.05 for the whole group and divided according to menopausal status, respectively; Table 4). We did not find any correlation between pelvic vein dilatation and other factors such as BMI, height, position of the uterus (ante- or retroverted), blood group, use of hormonal contraception, cigarette smoking or excessive consumption of alcohol (p>0.05). Degree of measured vascular dilatation was not correlated with the severity of pain-symptoms (p>0.05; Table 5). PCS symptoms, which have previously been described in the literature such as dyspareunia, ovarian point tenderness on bimanual examination and lower limb or vulvar varicosities, were not correlated with occurrence of CPP in our population.

**Table 4:** Characteristics not associated with PCS.

	Premenopausal			Postmenopausal		
	PCSG	CG1	CG2	PCSG	CG1	CG2
n	44	73	71	10	166	32
Age (SD)	37 (8)	39 (7)	35 (11)	54 (9)	66 (14)	62 (11)
BMI (SD)	26.5 (6.8)	26.5 (7)	31.5 (9.5)	29.2 (7.6)	25.2 (7.6)	24.3 (6.5)
Height (SD)	1.63 (0.07)	1.63 (0.09)	1.61 (0.1)	1.59 (0.07)	1.64 (0.07)	1.60 (0.04)
Gravida (SD)	3 (2)	3 (2)	1 (1)	4 (2)	1 (1)	3 (3)
Para (SD)	2 (1)	2 (1)	1 (1)	3 (2)	2 (1)	3 (3)

PCS: pelvic congestion syndrome

PCSG: pelvic congestion syndrome group

CG1: control group 1 (dilated ovarian or para-uterine veins, no chronic pelvic pain)

CG2: control group 2 (normal ovarian and para-uterine veins, no chronic pelvic pain)

SD: standard deviation

**Table 5:** Diameters of OV and PV.

Vein	Statistical function	Premenopausal			Postmenopausal		
		PCSG	CG1	CG2	PCSG	CG1	CG2
LOV (mm)	mean (SD)	7.2 (2)	7.3 (2.1)	2.8 (0.1)	8.3 (2.5)	8.1 (1.8)	3 (0.9)
	median	7.2	7.3	3.0	8.8	7.8	3.0
	max	11.7	14.2	5.0	13.1	12.6	5.1
ROV (mm)	mean (SD)	6.2 (1.6)	6.3 (1.7)	3 (0.9)	4.6 (2.2)	5.1 (1.9)	3.1 (0.7)
	median	6.2	6.4	3.0	6.3	5.2	3.0
	max	11.9	11.4	4.8	7.6	11.1	4.4
LPV (mm)	mean (SD)	4.6 (3.2)	4.7 (2.9)	-	5.6 (4.5)	4.5 (3.2)	-
	median	5.4	5.4	-	5.9	5.3	-
	max	10.0	10.9	-	9.8	5.3	-
RPV (mm)	mean (SD)	4.1 (3.1)	4.7 (3.1)	-	3.8 (2.1)	3.4 (2.9)	-
	median	5.1	3.9	-	3.9	3.9	-
	max	11.1	12.3	-	6.8	11.2	-

OV: ovarian veins

PV: para-uterine veins

LOV: left ovarian vein

ROV: right ovarian vein

LPV: left para-uterine vein

RPV: right para-uterine vein

SD: standard derivation

PCSG: pelvic congestion syndrome group

CG1: control group 1 (dilated ovarian and para-uterine veins, no chronic pelvic pain)

CG2: control group 2 (normal ovarian and para-uterine veins, no chronic pelvic pain)

**Fig 1:**
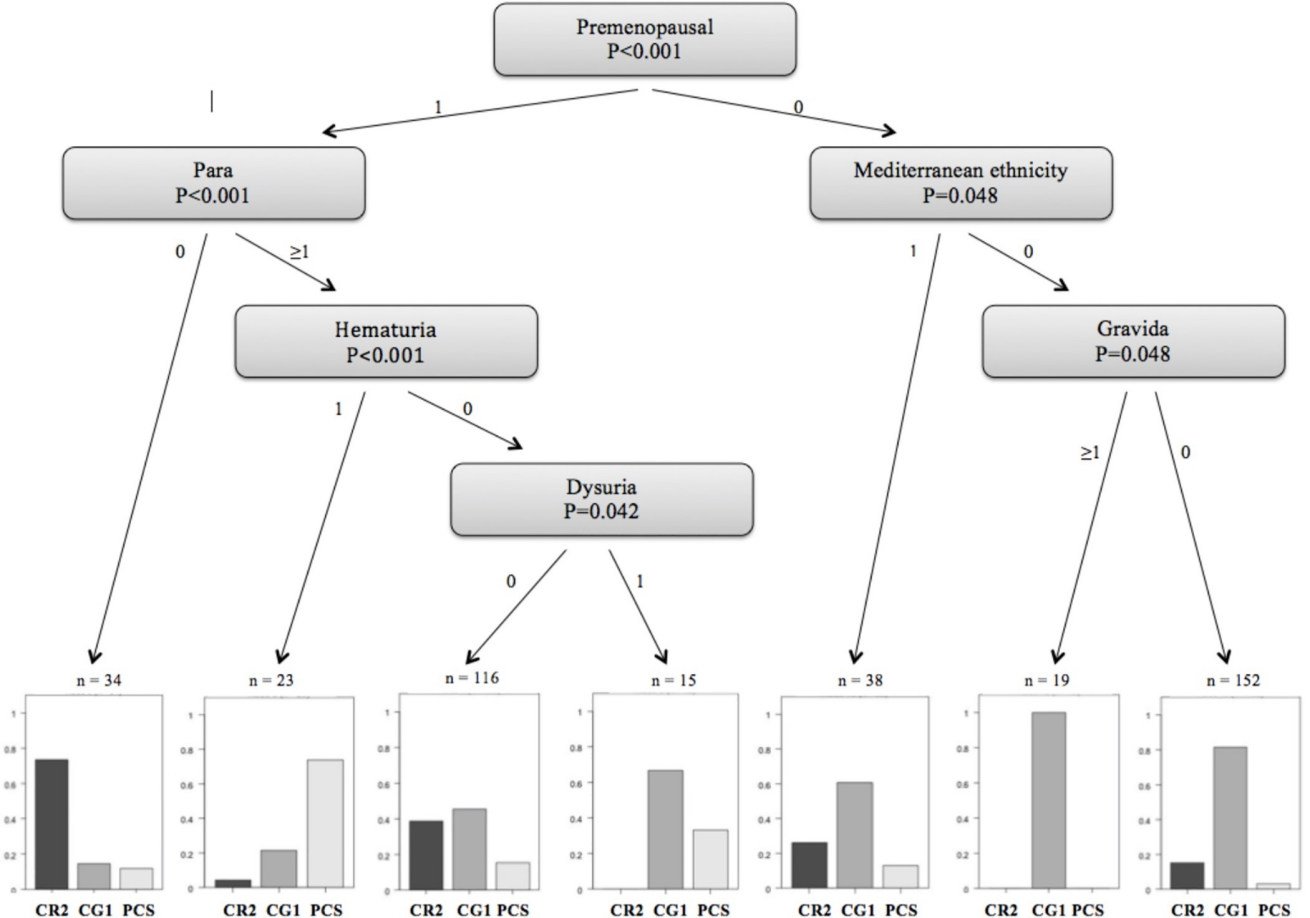
Predictors for PCS for women with CPP. PCS: pelvic congestion syndrome CPP: chronic pelvic pain PCSG: pelvic congestion syndrome group CG1: control group 1 (dilated ovarian or para-uterine veins, no chronic pelvic pain) CG2: control group 2 (normal ovarian and para-uterine veins, no chronic pelvic pain).

Based on a tree-based statistical model we found a strong positive correlation (80%) between CPP and dilated OV in premenopausal parous women with hematuria (Fig 1). These results suggest that a particular focus for the evaluation of PCS should be considered in cases of young parous patients with unexplained hematuria.

## Discussion

The purpose of this study was to investigate the prevalence and types of symptoms in patients with PCS using data from our large gynecological outpatient clinic. Therefore, we investigated the prevalence of CPP in women with dilated OV. We found this constellation in 2% of all investigated women. This result is lower than previously published data which showed that symptomatic dilated OV or PV occur in about 5.8% patients in a study of 273 healthy female kidney donors (9.9% women aged 18–76 years with pelvic varicosity, about 59% symptomatic cases) [15]. We further stratified our study group based on menopausal status. Interestingly, the incidence of CPP associated with dilated OV (PCS) rose to 8% (one out of every 12 women in our study collective) when only PreMP were taken into consideration. This result could support the theory that the etiology of PCS involves not only mechanical but also hormonal factors, and that ovarian activity contributes to the development of varicosities. One of the contributing factors to vascular distension is probably the relaxative venous effect of estradiol and estrone, which in OV are found at a 100-fold higher concentration than in the peripheral circulation [25]. Mechanical factors in the etiology of PCS include congenital absence [18] or acquired destruction of valves in the OV, inherited venous wall weakness, or increased venous pressure caused by mechanical obstruction, Valsalva maneuver, or effect of gravity in the upright position. That in turn may lead to venous pooling and pathological engorgement [26]. Pregnancy can further exacerbate this pathology [27].

In our study, PCS associated symptoms included previously described colicky lower abdominal, back or flank pain with accompanying hematuria, dysuria, and urinary frequency. There is no clear explanation for the pathophysiology of PCS-related pain. The current theory is that the ovarian venous engorgement results in the stretching of the intima, which consequently leads to the distortion of the endothelium and smooth muscle cells within the vessels. Consequently, vasoactive substances, including substance P (neuropeptide) and neurokinins A and B, are released, which contribute to inflammatory processes and pain. This may have a potential mass effect from enlarged pelvic veins leading to irritation of adjacent nerves [28].

The pain symptoms in our study collective were often accompanied by hematuria, dysuria, and urinary frequency. Hematuria has been mentioned in the literature as a symptom associated with the nutcracker syndrome [29]. This condition results in left renal venous hypertension, which leads to development of collateral veins with intra- and perirenal varicosis. It is believed that there is a direct communication between those venous vessels and the renal calyx, which explains the presence of hematuria [25]. As in our study, only five women with nutcracker syndrome contributed to the PCS group; hematuria in other patients could possibly be explained by renal venous hypertension due to reflux into OV. Presence of dysuria and urinary frequency in PCS patients with unremarkable urine samples, mimicking a urinary tract infection, has been described as a result of varicosis in the bladder trigone [5].

In our study the presence of above described symptoms was statistical significant for PCS. Furthermore, we could present a simple constellation of patient’s characteristics and symptoms (based on Fig 1) that allows for easier identification of women who might be suffering from PCS. The probability of PCS is high if the patient with CPP is premenopausal, gave a birth to at least one child and suffers from an otherwise inexplicable hematuria. In our opinion, especially those patients should receive a further radiological evaluation of the OV and, in case of diagnosed PCS, an appropriate treatment.

Other previously reported symptoms, above all dyspareunia and ovarian point tenderness, believed to be highly specific for PCS [4], did not show any correlation with this condition in our results. One possible explanation might be poor documentation in the records, with such symptoms being given less consideration compared to others. Vulvar and lower extremity varicosities, as well as chronic venous insufficiency of the lower limbs, were very rare in our study collective and did not show any correlation with PCS.

Asymptomatically dilated OV and PV were found in about 10% of all included patients. This result is higher than in the previously published data of Belenky et al. [15], where the prevalence was about 4% (out of 273 women), but lower than in the study of Koc et al. [16] with 18% (out of 324 women), and Rozenblit et al. [17] - 43% (34 PreMP only). We did not find any significant difference between PreMP (13%) and PMP (9%). As our data are derived from a larger patient cohort we believe that they may be more accurate.

It remains unknown why some patients with insufficient OV and PV suffer from pain while others do not. Interestingly, the degree of dilation did not show any correlation with the severity of pain. We can probably compare this phenomenon with chronic venous insufficiency of the lower extremities. Many varicose veins in the lower extremities do not cause pain and treatment is only indicated for those who are symptomatic [30].

We chose CT as the imaging method for our retrospective evaluation because of its wide availability and high number of abdominal and pelvic examinations (in comparison to MRI) performed for various indications (in order to include not only women with abdominal pain). Additionally, CT allows for exclusion of extrinsic causes of vein dilatation and is independent of examiner skills (in contrast to ultrasonography-based examinations). Its relatively low cost is a further advantage that makes CT a plausible option as an imaging modality to evaluate venous engorgement.

The cut off value of OV diameter of ≥ 6 mm that we used is based on numerous, well established and referenced MRI, CT and US based studies and has been widely accepted in the literature [8,16,17,20,21,22]. Although phlebography is considered to be the gold standard for diagnosis of venous incompetence, due to the nature of image acquisition in conventional angiography, the measurements are dependent on calibration and can vary significantly depending on the structure used as a reference as demonstrated by Jaskolka et al. [31]. Previous publications about OV and PV measurements acquired with conventional venography almost never mention the calibration method used to ensure reproducibility of results or to enable proper comparison with other imaging methods.

## Conclusions

In our study group as much as 8% of all premenopausal women had documented undiagnosed CPP correlated with dilated OV but not with vulvar and lower extremity varicosities. This underlines the common notion that identification of PCS patients admitted to gynecological outpatient clinics due to CPP still remains challenging and emphasizes the importance of including PCS in the differential diagnosis of CPP.

Taking into consideration that CPP is a common cause of gynecological referrals, awareness of PCS needs to be raised among physicians. Here we present for the first time a statistical significant constellation of patient’s characteristics and symptoms that allows for easier identification of patients who might be suffering from PCS. The probability is high if the patient with CPP is a premenopausal parous woman with unexplainable hematuria. The features we identified in this study as most relevant should enable a simplified recognition of women who can potentially profit from specific treatment that is available in the form of endovascular embolization. Moreover, we believe that the widely accepted cut-off value of 6 mm OV-diameter (with or without dilated PV) in imaging studies (such as CT in our case) can be reported by radiologists to at least raise the suspicion of PCS in the presence of CPP and lead to further clinical evaluation.
